# Sex-Specific Analysis of Quadriceps and Hamstring Strength Following Anterior Cruciate Ligament Reconstruction With or Without Meniscal Repair

**DOI:** 10.3390/jfmk11030318

**Published:** 2026-08-15

**Authors:** Christopher J. Cleary, Olivia Federico, Lexy N. Farrington, Nicholas S. Dombrowski, Christopher D. Bernard, Zachary D. Denton, Bryan G. Vopat, Ashley A. Herda

**Affiliations:** 1 Department of Health and Kinesiology, Bridgewater State University, Bridgewater, MA 02325, USA; 2 School of Medicine, University of Kansas Medical Center, Kansas City, KS 66160, USA; 3 Department of Orthopedic Surgery and Sports Medicine, University of Kansas Medical Center, Kansas City, KS 66160, USA; 4 Department of Health, Sport, and Exercise Sciences, University of Kansas, Lawrence, KS 66045, USA

**Keywords:** rehabilitation, symmetry, ratio, muscle, surgery

## Abstract

**Background and Objectives**: Strength deficits are extremely common following anterior cruciate ligament reconstruction (ACLR). These deficits may be further impacted by concomitant meniscus repair during ACLR. However, there remains limited evidence investigating the effect of meniscus repair on lower-limb strength after ACLR, and particularly if there are between-sex differences in strength after ACLR with and without meniscus repair. Therefore, the purpose of this study was to investigate sex-specific differences in meniscus repair during ACLR on quadriceps and hamstring isometric strength. **Methods**: Eighty-five (37 females; 19.0 ± 6.6 years old) patient charts were retrospectively evaluated after undergoing ACLR. Variables documented included meniscal status, maximal isometric quadriceps and hamstring strength of the operative (OP) and non-operative (NOP) legs. Three separate 3-way [leg (OP vs. NOP) × sex (males vs. females) × meniscus repair status (yes vs. no)] mixed-factorial analyses of variance (ANOVA) assessed differences in quadriceps and hamstring strength, and the hamstring-to-quadriceps strength ratio (H:Q), at an alpha level of *p* ≤ 0.05. **Results**: For H:Q there was a sex by meniscus repair status interaction (*p* = 0.007). Females who underwent meniscus repair had lower H:Q (18.2%) than those without. Females without meniscus repair had a 20.3% greater H:Q than males without meniscus repair. Males had greater quadriceps strength than females (mean difference ± standard error; 0.58 ± 0.28 N∙kg^−1^) and the NOP leg was stronger than OP for quadriceps (0.34 ± 0.08 N∙kg^−1^) and hamstring (0.14 ± 0.04 N∙kg^−1^) strength across sex. **Conclusions**: These findings suggest that the H:Q ratio differed according to sex and meniscus repair status. Further, there was a between-limb difference for strength in the quadriceps and hamstrings along with sex-related differences in strength. However, meniscus repair status did not influence raw strength values.

## 1. Introduction

It is estimated that approximately 350,000 individuals undergo anterior cruciate ligament (ACL) reconstruction (ACLR) surgery each year in the United States [1,2]. ACL injuries are frequently accompanied by concurrent meniscus, cartilage, and collateral ligament injuries [3,4], which require concomitant procedures during surgery. Examples of concomitant procedures are meniscal resections or repairs, collateral repairs, chondral procedures, osteotomies, and patellar procedures [5]. These concomitant procedures highlight the complexities of ACL injuries and may contribute to additional post-surgical muscular deficits in the thigh musculature altering post-operative recovery.

According to US registry data from 2010 to 2018, approximately 50% of all patients undergoing ACLR had a concomitant meniscal procedure. [6]. Post-operative musculoskeletal outcomes and rehabilitation protocols following meniscal repair may differ substantially from ACLR without meniscal repair [7,8], which may lead to altered musculoskeletal outcomes. For example, a vital outcome after ACLR is the maximal strength of the quadriceps and hamstrings, commonly measured through isometric tasks to determine patient readiness to return to sport [9,10]. Strength deficits of the lower limb, particularly in the operative limb compared to the non-operative limb, have been extensively reported after ACLR [11,12,13]. Further, the ratio of hamstring strength to quadriceps strength (H:Q), is a common clinical variable of interest during rehabilitation [14]. For example, it is thought that a lower H:Q ratio may indicate within-leg asymmetries for muscle function, which could predispose an individual to future injuries. These between-limb strength deficits after ACLR are postulated to be due to a myriad of physiological factors [12,15,16,17] that are beyond the scope of the current paper’s rationale.

Given the complexity of ACL injuries and the ACLR post-operative recovery process, determining if meniscal repair, concomitantly performed with ACLR surgery, alters post-operative muscle strength deficits is necessary. Further, due to the overall underrepresentation of female athletes in sports medicine, sports science, and ACLR research [18,19], sex-specific differences in muscle strength after ACLR with and without meniscus repair should be considered. Sex-specific differences in neuromuscular characteristics, force production, kinematics, and sport performance in healthy populations have been highlighted in recent randomized control trials [20,21,22] and reviews [23]. Therefore, any of the documented sex-based differences in strength [12,24,25,26] after ACLR may not be solely attributed to the injury or surgery themselves but an inherent biological difference in skeletal muscle and/or physiological characteristics between males and females. Regardless, the number of females experiencing ACL injuries and undergoing ACLR is continuing to rise, without a concomitant increase in females being included in physical performance research [19]. Thus, studies that report sex-specific musculoskeletal outcomes regarding ACLR, particularly with and without meniscus repair, remain imperative [26].

This retrospective observational study aimed to investigate the sex-specific differences in concurrent meniscal repair during ACLR on quadriceps and hamstring strength and the H:Q ratio. It was hypothesized that the group with meniscus repair would have worsened quadriceps and hamstring strength than the group without and that females would be weaker than their male counterparts. With the findings of this study, practitioners will be able to optimize post-surgical treatment strategies for their patients who suffer from ACL and meniscal injuries.

## 2. Materials and Methods

### 2.1. Study Design

This study was conducted as a retrospective chart review which included male and female patients that had undergone ACLR by one of four orthopedic surgeons associated with the University hospital system between 2018 and 2022. Charted variables of interest were de-identified and included isometric quadriceps and hamstring strength of both legs, patient descriptive characteristics, and surgical procedures. Functional assessments were performed during the patient’s return to sport (RTS) testing visit conducted by a physical therapist and occurred approximately 5–8 months after ACLR. This study was conducted in accordance with the Declaration of Helsinki and subsequently approved as a retrospective chart review by the University of Kansas Medical Center Human Research Protections Program; #STUDY00148827. The Human Research Protections Program determined that informed consent was not needed for this retrospective chart review and all data were de-identified prior to analyses.

### 2.2. Subjects

This retrospective chart review included data from 85 patients (48 males, 37 females; 19.0 ± 6.6 years old, 6.6 ± 1.7 months post-ACLR) that were evaluated after undergoing primary ACLR with and without meniscus repair. Patients’ charts were extracted from the University-affiliated hospital database using the Current Procedural Terminology (CPT) code for arthroscopically aided ACL repair/augmentation or reconstruction (CPT 29888) between 2018 and 2022. Extracted data (accessed between 12–16 December 2022) included the following: patient biological sex, surgical report data, age, date of surgery, date of RTS testing, and strength testing results. From this population, any patient that had undergone previous ACLR, conducted RTS testing through a clinic not associated with the hospital system in which their surgery was performed, underwent a meniscus procedure other than meniscus repair (e.g., meniscectomy) or did not perform RTS testing were removed from analyses. No exclusions were placed on age, sex, pre-surgery activity level, sport participation, or graft type/location.

### 2.3. Outcome Measures

According to patient charts, approximately 5–8 months after ACLR, patients underwent a functional performance assessment to determine physical readiness to return to sport. This 5–8 month timeline is the standard functional performance testing date for the University-affiliated sports medicine clinics. Prior to the functional performance assessment, all participants underwent a standardized rehabilitation protocol consisting of twice-weekly physical therapy sessions lastly approximately 45–60 min per session at a University-affiliated sports medicine clinic. Detailed information regarding this rehabilitation protocol can be found online (https://www.kansashealthsystem.com/care/treatments/physical-therapy/resources, accessed on 1 April 2026). The exercises utilized are similar in cases of ACLR with meniscus repair and without; however, the timelines of progression do differ slightly as is noted in the appropriate rehabilitation protocols linked above. Briefly, the protocol consisted of five phases in which phase 1 began post-surgery and included basic quadriceps “activation” exercises (e.g., “quad sets”) exercises with neuromuscular electrical stimulation if necessary. Phase 2 introduced bilateral squats, step-up/step-down exercises, and leg press and hamstring curls in the later portion of Phase 2. Some of the exercises included in phase 3 were weighted squats, lunges, single-leg stability movements, and basic linear treadmill running. Phase 4 introduced low-intensity box jumps, more intensive squats and lunges, and leg extensions. Lastly, phase 5 incorporated sports-specific drills and more intensive plyometric progressions.

For the current study, the primary outcome measures recorded from the patient charts included maximal voluntary isometric contractions (MVICs) during knee flexion and extension to represent hamstring and quadriceps strength, respectively, using a belt-stabilized handheld dynamometer (microFET 2, Hoggan Scientific LLC., Salt Lake City, UT, USA). The non-operative (NOP) leg was assessed first for each patient followed by the operative (OP) leg. During MVICs, patients were seated upright with the knee flexed at ~90° and the dynamometer stabilized on the distal leg. Knee extension MVICs assessed quadriceps strength while knee flexion MVICs measured hamstring strength. Patients performed three 3 s MVICs, with the highest value (kg) recorded and subsequently converted to Newtons (N). The H:Q ratio was calculated for each leg as the ratio of hamstrings MVIC to quadriceps MVIC. All MVIC data were expressed relative to the participant’s body mass (N∙kg^−1^ of body mass) to ensure accurate comparison between sexes [20,21,22]. A knee angle of ~90° is frequently utilized after ACLR for both knee extension and knee flexion testing [9,27] despite not being an optimal angle for maximal force production. Further, handheld dynamometry for isometric strength is increasingly utilized in clinical settings in which a “gold standard” isokinetic dynamometer is not readily available.

### 2.4. Statistical Analyses

An independent samples *t*-test was utilized to determine if there were differences between groups in time from surgery to performance testing date. To assess differences in quadriceps strength, hamstring strength, and the H:Q between leg, sex, and meniscus repair status, three separate, 3-way [leg (OP vs. NOP) × sex (males vs. females) × meniscus repair status (yes v. no)] mixed factorial analyses of variance (ANOVA) were conducted. In the case of a significant 3- or 2-way interaction, ANOVAs were decomposed into lower-order ANOVAs and main effects were followed up with paired or independent samples *t*-tests. Mean differences are presented as mean difference ± standard error (SE) with corresponding 95% confidence intervals (CI) of the mean differences and Cohen’s *d* as a measure of effect size. All data were considered significant at an alpha level of *p* < 0.05. All analyses were performed in SPSS (version 29, IBM, Armonk, NY, USA).

## 3. Results

Of the 85 patients included in the current study, 45 underwent meniscus repair during ACLR (21 females; 17.7 ± 3.4 years, 6.8 ± 1.4 months post-ACLR). The remaining 40 patients (16 females; 20.5 ± 8.7 years; 6.4 ± 2.0 months post-ACLR) did not undergo meniscus repair. There was no difference in time from surgery to performance testing date between the two groups (*p* = 0.337). In the meniscus repair group, 34 of the patients received a bone–patellar tendon–bone (BTB) graft (allograft: *n* = 2, autograft: *n* = 32), 10 received a hamstrings graft (allograft: *n* = 1, autograft: *n* = 9), and one patient received a peroneal allograft. Lastly, in the non-meniscus repair group, 29 patients received a BTB graft (allograft: *n* = 2; autograft: *n* = 29) and 11 received a hamstring graft (allograft: *n* = 1; autograft: *n* = 10).

The ANOVA models for the hamstring and quadriceps strength indicated no significant 3- or 2-way interactions (Table 1; *p*-value range: 0.218–0.719). There was also no main effect for MSR for in quadriceps (*p* = 0.828, *d* = 0.03) and hamstring strength (*p* = 0.950, *d* = 0.009). There was no main effect for sex in the hamstrings (*p* = 0.094). However, there was a main effect for sex in the quadriceps, as the males were stronger than the females (*p* = 0.038; mean difference ± standard error = 0.58 ± 0.28 N∙kg^−1^; 95% CI of the mean difference: 0.032 N∙kg^−1^ to 1.13 N∙kg^−1^; *d* = 0.327). Further, there was a main effect for leg in both the quadriceps and hamstrings, as the NOP limb was stronger than OP for the quadriceps (*p* < 0.001; 0.34 ± 0.08 N∙kg^−1^; 95% CI: 0.175 N∙kg^−1^ to 0.509 N∙kg^−1^; *d* = 0.635) and hamstrings (*p* = 0.002; 0.141 ± 0.04 N∙kg^−1^; 95% CI: 0.053 N∙kg^−1^ to 0.228 N∙kg^−1^; *d* = 0.498).

For H:Q, there was no 3-way interaction (*p* = 0.991). A 2-way interaction for sex × meniscus repair status (*p* = 0.007) revealed that females with meniscus repair had lower H:Q (*p* = 0.031; 0.13 ± 0.06; 95% CI: 0.012 to 0.251; *d* = 1.17) than those without, and females without meniscus repair had higher H:Q than males without repair (*p* = 0.016; 0.14 ± 0.06; 95% CI: 0.028 to 0.260; *d* = 1.28). H:Q was otherwise similar between sex for patients who had meniscus repair (*p* = 0.159; *d* = 0.683) and males had similar H:Q regardless of their meniscus repair status (*p* = 0.091; *d* = 0.793). No additional 2-way interactions for H:Q (*p*-value range = 0.835–0.861) or main effect for leg (*p* = 0.201) were indicated.

## 4. Discussion

This study aimed to evaluate the impact of concurrent meniscus repair alongside ACLR on isometric strength of the quadriceps and hamstrings in males and females. The primary findings indicated meniscus repair had no effect on absolute maximal isometric strength. However, when the data were expressed as a ratio (H:Q), females without concurrent meniscus repair had a higher H:Q than females with meniscus repair by 18.2%. Further, when comparing patients without meniscus repair, females had a 20.3% greater H:Q than males. Irrespective of meniscus repair or sex, the NOP leg was 8.4% and 4.6% stronger than OP for the quadriceps and hamstrings, respectively. Males also had greater quadriceps strength than females by 13.8% and when leg and surgical status were combined without a sex difference in hamstring strength.

Between-limb asymmetries in maximal quadriceps [12,28,29] and hamstring [11,30,31] isometric strength following ACLR has been extensively documented. The finding of the present study adds to this documentation, as maximal quadriceps and hamstring strength in the OP leg was significantly weaker than the NOP leg, independent of meniscus repair status and sex. The sample of the present study was on average 6.6 ± 1.7 months post-ACLR and there was an 8.4% and a 4.6% difference between the OP and NOP legs for quadriceps and hamstring MVIC strength, respectively. This finding of a post-ACLR strength deficit is in line with similar populations at 6 months post-ACLR [29,32].

Although the magnitude of the between-limbs strength deficit in the hamstrings was less than that of the quadriceps, there was still a 4.6% difference. Collectively, these isometric strength findings add to the ever-growing evidence of weakened strength after ACLR and rehabilitation. Future research should continue to investigate factors that may influence this strength deficit, including but not limited to, muscle size [33,34], subjective psychological variables [35], and neuromuscular-related characteristics [17,36]. Another primary finding of this study was that meniscus repair had no effect on quadriceps or hamstrings isometric strength, which is in line with previous research [8,37,38]. However, when the strength data were expressed as a ratio of hamstring strength to quadriceps strength, the H:Q, the results were not as clear. Despite the lack of between-limb differences in H:Q in either sex or group evaluated, the H:Q was larger in females without meniscus repair than those with by 0.13 (18.2% difference), when legs were combined. This finding suggests that meniscus repair may have a negative effect on this particular strength ratio, but in females only. However, it is important to note that the impact of the H:Q on future injury risk and performance after ACLR remains relatively unknown [39]. From a physiological perspective, these between-limb strength deficits may be due to—but not limited to—factors such as reduced muscle size [40,41], altered neuromuscular system activity [15,36], and cellular activity [16,34].

Following rehabilitation, the OP demonstrated lower MVIC strength than the NOP leg, which was to be expected. However, the inclusion of meniscus repair in conjunction with ACLR did not compound muscle strength deficits following surgery although there were slight differences in H:Q. This study and others [24,25,42] indicated sex-specific differences in limb strength, where females had lower absolute quadriceps and hamstring strength than the males. Conversely, in the group that underwent meniscus repair during ACLR, females had a greater H:Q than males, suggesting a more comparable strength balance in females with meniscus repair than males, collapsed across legs. However, a novel finding was that females who did not undergo meniscus repair with ACLR had a larger H:Q than females who did, suggesting a potential sex-specific response of meniscus repair on strength ratios which was not observed for the males and warrants further research. These unique findings indicate that sex-related differences in absolute isometric strength are reduced with ratio data. This is particularly evident when the ratio data are analyzed through sub-group analyses (sex and meniscus repair status), which is a novel aspect of the current study. Therefore, the additional impact of ACLR plus meniscal repair on this ratio may require additional rehabilitative intervention protocols for this procedure specifically. Potentially, these sex-related differences in muscular strength may be due to factors not investigated in the present study, such as contractile properties [43], muscle architecture [44], and motor unit control strategies [45,46]. The influence of these factors on sex differences in muscular strength post-ACLR should be investigated in future research.

A unique and novel aspect of this study is the evaluation of sex-specific strength outcomes in combination with meniscus repair status. As recently documented by Crowell et al., females are overall underrepresented in the literature on ACLR rehabilitation and represent only approximately 45% of all studies [19]. With the heightened participation of females in sports [47] and the high rates of ACL tears in females, particularly young females [48], the inclusion of females in ACL studies is paramount. Apparent limitations of this study include the small sample size, retrospective design, and the absence of pre-operative strength measurements. Only the assessment of isometric strength was retrieved from charts; therefore, any conclusions on the effect of meniscus repair status on other functional outcomes after ACLR (e.g., vertical jump height, hop distance, neuromuscular assessments) cannot be made from these data. Subsequently, any variables that are sometimes concurrently assessed with isometric strength (e.g., rate of force development) were not evaluated in the present study due to the clinical use of a handheld dynamometer rather than a more-sensitive isokinetic dynamometer chair. Lastly, torque could not be calculated in the present study as lever-arm length was not measured. Therefore, anthropometric differences might be partially responsible for the results observed in the present study.

## 5. Conclusions

Practically, the present findings suggest that there may always be apparent absolute sex and leg differences in maximal isometric strength following ACLR. However, sex and leg differences may not be as apparent with common clinical ratios, such as the H:Q. Thus, practitioners and clinicians should continually evaluate the ‘raw’ strength and ratio strength of the involved musculature after orthopedic-related surgeries such as ACLR to evaluate the post-surgical musculoskeletal outcomes along with potential future injury risk and readiness to return to sport following injury and surgery. Lastly, the observed findings might suggest that individualized rehabilitation programs, based on sex and meniscus repair status may be warranted after ACLR. As the participants in the current study all underwent a standardized rehabilitation protocol, it may be possible that individualized protocols are necessary to overcome strength deficits post-ACLR. However, this warrants further extensive further research.

## Figures and Tables

**Table 1:** Between-sex, leg, and meniscus repair status differences for quadriceps strength, hamstring strength, and H:Q.

	Males (*n* = 48)		Females (*n* = 37)		Overall (*n* = 85)	
	No MSR (*n* = 24)	MSR (*n* = 24)	No MSR (*n* = 16)	MSR (*n* = 21)	No MSR (*n* = 40)	MSR (*n* = 45)
Quadriceps						
OP (N∙kg^−1^)	4.48 ± 1.22 *	4.26 ± 1.29 *	3.62 ± 1.17	3.90 ± 1.25	4.14 ± 1.26	4.09 ± 1.27
NOP (N∙kg^−1^)	4.85 ± 1.26 *^#^	4.51 ± 1.32 *^#^	3.87 ± 1.31 ^#^	4.40 ± 1.58 ^#^	4.46 ± 1.36	4.46 ± 1.43
Hamstring						
OP (N∙kg^−1^)	3.21 ± 1.01	3.34 ± 1.04	3.00 ± 0.86	2.76 ± 1.00	3.13 ± 0.95	3.07 ± 1.05
NOP (N∙kg^−1^)	3.33 ± 0.98 ^#^	3.45 ± 1.11 ^#^	3.09 ± 0.82 ^#^	3.02 ± 1.02 ^#^	3.23 ± 0.91	3.25 ± 1.08
H:Q						
OP	0.72 ± 0.16	0.81 ± 0.21	0.86 ± 0.21 ^†§^	0.73 ± 0.23	0.78 ± 0.19	0.77 ± 0.22
NOP	0.69 ± 0.15	0.79 ± 0.23	0.84 ± 0.20 ^†§^	0.71 ± 0.18	0.75 ± 0.19	0.75 ± 0.21

* main effect for sex collapsed across legs and MSR; ^#^ main effect for leg collapsed across sex and MSR; ^†^ difference between males and females without MSR collapsed across legs; ^§^ difference between females with and without MSR collapsed across legs; H:Q: hamstrings to quadriceps ratio; MSR: meniscus repair; N∙kg^−1^: Newtons of MVIC force per kilogram of body mass.

## Data Availability

The raw data supporting the conclusions of this article will be made available by the authors on request.

## Abbreviations

The following abbreviations are used in this manuscript:

ACL	Anterior cruciate ligament
ACLR	Anterior cruciate ligament reconstruction
ANOVA	Analysis of variance
CPT	Current procedural terminology
H:Q	Hamstrings to quadriceps ratio
MVIC	Maximal voluntary isometric contraction
N	Newtons
NOP	Non-operative limb
OP	Operative limb
RTS	Return to sport
